# Effects of Electroacupuncture Treatment on Bone Cancer Pain Model with Morphine Tolerance

**DOI:** 10.1155/2016/8028474

**Published:** 2016-09-08

**Authors:** Lei Sima, Bifa Fan, Longtao Yan, Yuan Shui

**Affiliations:** National Pain Management and Research Center, China-Japan Friendship Hospital, Beijing, China

## Abstract

*Objective*. To explore the efficacy of electroacupuncture treatment in cancer induced bone pain (CIBP) rat model with morphine tolerance and explore changes of calcitonin-gene related peptide (CGRP) expression in dorsal root ganglion (DRG).* Methods*. Forty SD rats were divided into five groups: sham, CIBP (B), CIBP + morphine (BM), CIBP + electroacupuncture (BE), and CIBP + morphine + electroacupuncture (BME). B, BM, BE, and BME groups were prepared CIBP model. The latter three groups then accepted morphine, electroacupuncture, and morphine combined electroacupuncture, separately, nine days consecutively (M1 to M9). Mechanical withdraw threshold (MWT) was evaluated.* Results*. BE group only had differences in M1, M2, and M3 compared to B group (*P* < 0.01). From M5, BM group showed significantly decreased MWT. Electroacupuncture could obtain analgesic effects only at early stage (M1 to M5). From M5 to M9, BME had the differences with BM group (*P* < 0.01). IOD value of CGRP in BM and BME was substantially less than in B group. CGRP in BME was significantly lower than that in BM group (*P* < 0.01).* Conclusion*. When used in combination with electroacupuncture, morphine could result in improving analgesic effects and reducing tolerance. CGRP may be associated with pain behaviors.

## 1. Introduction

The incidence of bone metastases from commonly found malignant tumors including lung, breast, and prostate cancers is as high as 30–40%, 65–75%, and 65–75% [1], respectively. As there are rich sensory nerves on the periosteum, the invasion of the tumor to the periosteum will cause severe nociceptive pain, while the damaged sensory nerves could be further sensitized and remodeled, which could result in neuropathic pain [2]. Therefore, cancer induced bone pain (CIBP) includes both nociceptive pain and neuropathic pain. So the mechanism has been considered very complex. Currently, the cancer pain models used for the investigations mainly focused on the bone metastases. Opioids that are represented by morphine are the most used and most effective drug for the treatment of cancer pain; however, the use of morphine is greatly constricted by the high incidence of morphine tolerance. In addition, clinical practices show that patients with bone cancer pain are easier to develop morphine tolerance, which has been considered as one of the major causes of uncontrollable pain in the patients with advanced cancers [3].

*μ*-Opioid receptor (MOR) could not be endocytosed to allow desensitization and resensitization, which is associated with the downregulation of the MOR numbers on the cellular membrane [4]. The endocytosis of the MOR is mediated by the neurotransmitters including calcitonin-gene related peptide (CGRP) and substance P (SP), which play important roles in the negative regulation of the opioid tolerance [5]. Treating cancer pain with acupuncture has been recommended by the NCCN cancer treatment guideline, the most used guideline for the treatment of tumors all over the world, issued by the National Cancer Institute (NCI) of USA. In recent years, treating cancer pain [6, 7], especially the bone cancer pain, with acupuncture has provided a lot of valuable evidence [8, 9]. However, no study focused on the effects of electroacupuncture treatment on bone cancer pain-morphine tolerance has been published to date.

## 2. Materials and Methods

### 2.1. Animals and Equipment

Prior permission for animal experimentation was obtained from the Institutional Animal Ethics Committee. Forty SPF grade female SD rats with the body weights of 180–201 g were obtained from the Vital River Experimental Animal Center (Beijing, China, number SCXK9 (Jing) 2007-0001). Walker 256 breast cancer cells were obtained from the cancer institute of the Peking Union Medical College. Morphine was purchased from the first pharmaceutical company of Shenyang. Polyclonal rabbit anti-CGRP antibody (1 : 5000) was obtained from DAKO. Von Frey fibre was from Noah Coast Medical Company (USA). Han's electroacupuncture apparatus (LH202) with the parameters of 2/100 Hz and 1 mA was used.

### 2.2. Inducing the Bone Cancer Pain-Morphine Tolerance Rat Models

The rat model of bone cancer pain-morphine tolerance was induced according to the methods described in previous studies. In brief, intraperitoneal injection of Walker 256 breast cancer cells was performed for the rats, and then the ascites was collected on day 7 after the injection. The cell density was adjusted to 4 × 10^2^/*μ*L; thermal-inactivated cancer cells were also prepared. Intrathecal catheterization was performed for the rats, and lidocaine was used to evaluate whether the catheterization was successful.

The rats successfully catheterized were divided into 5 groups on day 5 after the catheterization using the random number table in each as follows: sham group, bone cancer pain group (B group), bone cancer pain plus morphine tolerance group (BM group), bone cancer pain plus electroacupuncture group (BE group), and bone cancer pain plus morphine tolerance plus electroacupuncture group (BME group). Tibia cancer pain model was induced for the rats in the B, BM, BE, and BME groups by injecting 10 *μ*L of human Walker 256 breast cancer cells into the bone marrow cavity at the upper part of the right tibia.

Our previous studies have demonstrated that bone cancer pain would occur on day 6 after the induction; thus, since day 7 after the induction, intrathecal injection of morphine (20 *μ*g/kg, 2 times/d, for 9 continuous days) for the rats in the BM group, electroacupuncture treatment for the rats in the BE group, and morphine plus electroacupuncture treatment for the rats in the BME group were performed, respectively, while, for the rats in the sham group, the upper part of the right tibia was exposed and intrathecal injection of the same volume of normal saline was performed since day 7.

### 2.3. Treatments

Zusanli (ST36) and Sanyinjiao (SP6) acupoints that were commonly used for the treatment of cancer pain were selected in this study [10]. For the rats in the BME and BE groups, 2/100 Hz high/low frequency alternating current Han's electroacupuncture apparatus was used for the treatment, with the stimulation intensities of 0.5, 1, and 1.5 mA that were increased every 10 min. The stimulation time was 30 min for each stimulation intensity. The treatment was started on day 7 after the induction with the frequency of 1 time/day for 9 continuous days.

### 2.4. Measuring the Pain Behaviors

The mechanical withdrawal threshold (MWT) of the rats was measured before the injection of the cancer cells (baseline, T0), at 3 and 6 days after the injection (T3 and T6), and at 1, 3, 5, 7, and 9 days after the morphine/electroacupuncture treatments (M1, M3, M5, M7, and M9). For von Frey testing, the rats were placed in enclosures on an elevated wire mesh floor, and mechanical allodynia was assessed using a dynamic plantar aesthesiometer (Ugo Basile, Stoelting, IL, USA). A metal probe (0.5 mm diameter) was directed against the hind paw pad, and an upward force was exerted. The force required to elicit a withdrawal response was measured in grams and automatically registered when the paw was withdrawn or the preset cut-off was reached (15 g).

### 2.5. Measuring CGRP Expression in the Dorsal Root Ganglion by Immunohistochemical Examinations

After the last measurement of the pain behaviors, the rats were anaesthetized with pentobarbital injection (100 mg/kg) and transcardially perfused with 4% paraformaldehyde in 0.1 M phosphate buffer saline (PBS). The dorsal root ganglion (DRG) was dissected out and preserved in PBS for a further period of 3 days. Cryostat sections (20 *μ* thick) were cut in the transverse plane at −20°C and collected as free-floating sections in 0.1 M phosphate buffer saline (PBS). An average of 11-12 sections per rat was collected. Later, the sections were processed for immunohistochemical localization of CGRP. Following this, sections were directly exposed to primary antibody against CGRP (1 : 400; Calbiochem, USA) for 48 h. CGRP expression was visualized by the ABC method (Vector Laboratories, USA) using 3,3-diaminobenzidine (DAB) tetrachloride (DAB) as the chromogen. Finally, the sections were taken on slides and dehydrated followed by clearing.

Images of the stained sections were captured under the microscope and the images were saved in ProgRes Capture Pro 2.7 Image analysis software (Jenoptik, Germany). The area of the CGRP expression in these sections, which corresponded to the superficial part of the DRG, was selected by Image Pro Plus software to calculate the integral optical density (IOD).

### 2.6. Statistical Analysis

SPSS 11.0 software was used for the statistical analysis. All the data were described as means ± standard divisions. One-way analysis of variance (ANOVA) and* t*-test were used for the comparisons. *P* < 0.05 was considered statistically significant.

## 3. Results

### 3.1. MWT of the Rats

**Figure 1:**
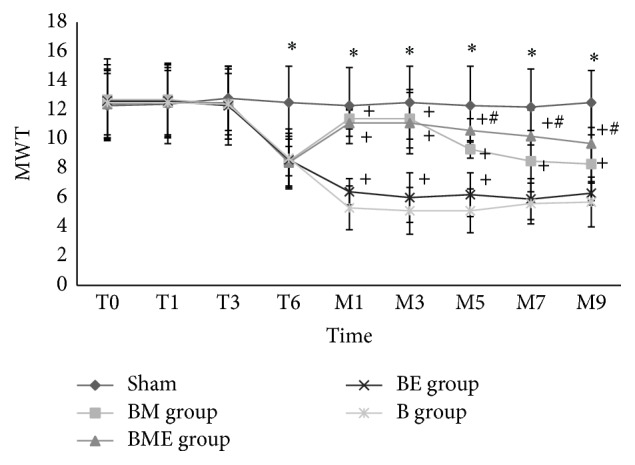
MWT of the rats during bone cancer pain and treatment. ^*∗*^Significant difference from sham group since day 6 (*P* < 0.01). ^+^Significant difference from B group since morphine/EA treatment (*P* < 0.01). ^#^Significant difference from BM group since morphine tolerance (*P* < 0.01).

On day 6 (T6) after the injection of the cancer cells, the MWT in all the four groups of the rats decreased significantly due to the CIBP caused by bone cancer, and the differences with the sham group were statistically significant (*P* < 0.01). From M1 to M9, all the groups with treatment had the differences compared with B group (*P* < 0.01). BE group only had differences in M1, M2, and M3 compared to B group (*P* < 0.01). From M5, BM group showed significant decreased MWT due to morphine tolerance. ED_50_ value was 70.4 ± 9.5 *μ*g for cumulative dose after morphine tolerance. Electroacupuncture treatment alone for bone cancer pain could obtain analgesic effects only at early stage (M1 to M5). But the analgesic effect was much less than morphine. The longer electroacupuncture treatment (M7 to M9) showed analgesic tolerance too. From M5 to M9, BME group had differences compared with the BM group (*P* < 0.01); see Table 1 and Figure 1.

### 3.2. CGRP Expression and the IOD Value

**Figure 2:**
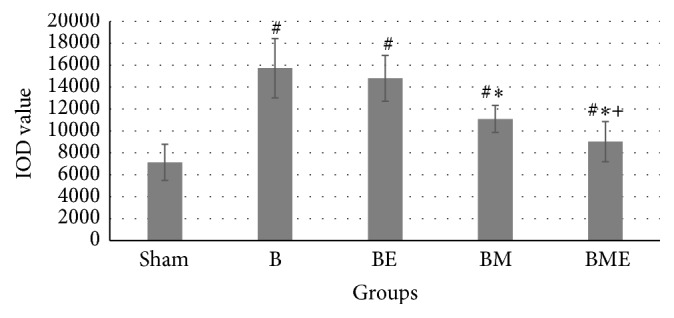
IOD value of CGRP in dorsal root ganglion of the rats. ^#^Compared with sham, *P* < 0.01. ^*∗*^Compared with B group, *P* < 0.01. ^+^Compared with BM group, *P* < 0.01.

**Figure 3:**
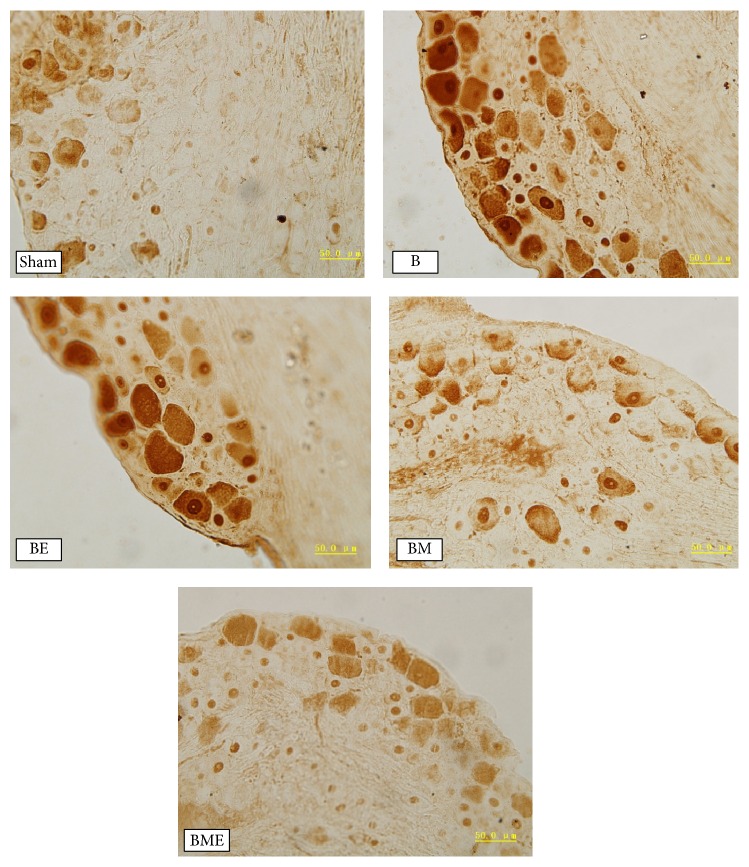
Expression of CGRP in dorsal root ganglion of the rats (CGRP staining, ×100).

The immunoreactive substances of CGRP peptide are mainly at the margins of the DRG, while the staining of the CGRP in B group and BE group was evidently deeper than B group. The IOD value of CGRP in BM and BME group was substantially less than in B group. The IOD value of the CGRP in the BME group was significantly lower than that in BM group (*P* < 0.01), as shown in Figures 2 and 3.

## 4. Discussion

According to the theories of zangfu and meridians, masters of traditional Chinese medicine proposed the theory of “Qi and blood running” to explain pain, which suggests “stagnation leading to pain.” The principle of acupuncture analgesia is soothing the channels and improving collateral circulation. Western medicine suggests that “acupuncture could result in analgesic effects via stimulating the release of endogenous opioid peptides,” and further suggests that stimulation at low frequency (2 Hz) could accelerate the synthesis and release of encephalin and endorphin, while stimulation at high frequency (100 Hz) could promote the synthesis and release of dynorphin. Stimulation with high-frequency electroacupuncture (2/100 Hz) could accelerate the synthesis and release of all these three opioid peptides [11–13]. The encephalin, endorphin, and dynorphin are the endogenous ligand for *μ*-, *δ*-, and *κ*-opioid receptor, respectively [14].

The findings of the present study showed that the pain behavior changes since day 6 after the injection of cancer cells, which was in agreement with the time reported in previous studies. Our findings showed that, after injection of morphine for 5 consecutive days, the MWT of the rats decreased, which suggested the occurrence of bone cancer pain-morphine tolerance. We speculated that the mechanisms involved could be associated with the overrelease of the SP and CGRP in the DRG. With more pain causing neurotransmitters release from the presynaptic membrane into the synaptic cleft, the analgesic effects of morphine reduced substantially.

In addition to this, the present study further demonstrated that electroacupuncture treatment is effective in treating morphine tolerance. The analgesic effects of using electroacupuncture treatment alone in the early stage of analgesia (M1–M3) were lower than using morphine alone, while the effects of using morphine plus electroacupuncture treatment were similar to using morphine alone, suggesting that the analgesic effects from morphine were dominant in the early stage of analgesia. However, morphine tolerance occurred in the late stage of analgesia (M5–M9), while the tolerance in the BME group was lower than in the BM group, suggesting that electroacupuncture treatment could effectively reduce the morphine tolerance and increase the MWT.

Previous studies have shown that electroacupuncture treatment could inhibit the thermal withdrawal response in the inflammatory pain-morphine tolerance models, and the underlying mechanisms are associated with the downregulation of the vanilloid receptor in the DRG and the inhibition of the expression of the CGRP and SP mRNA in the DRG [15]. CGRP mainly exists in the small or medium neurons in the DRG and is the most important neurotransmitter for the transmission of pain signals. However, using CGRP receptor antagonist CGRP8-37 could inhibit and reverse the morphine tolerance.

The findings of the present study showed that the effects of electroacupuncture treatment were similar to the application of CGRP8-37. We speculated that the mechanisms involved in the effects of electroacupuncture treatment on reducing the morphine tolerance were associated with the inhibition of the overrelease of CGRP in the DRG. Some other studies have also shown that electroacupuncture treatment could induce the release of endogenous opioid peptides, which participated in the phosphorylation and desensitization of *μ*-receptor in neuropathic pain, thus inhibiting the occurrence of morphine tolerance [16].

This study found some evidences. First, electroacupuncture treatment alone for bone cancer pain could obtain analgesic effects only at early stage. The longer electroacupuncture and morphine treatment may lead to analgesic tolerance. Second, when used in combination with electroacupuncture, morphine treatment could result in improving the analgesic effects and reducing the tolerance. Third, the expression of CGRP in the DGR may associate with the pain behaviors.

Of course, we need more proof to support the correlative association between the electroacupuncture and the effects on CGRP. More studies are needed to further investigate inhibitor of CGRP in the electroacupuncture group to confirm the effect of CGRP for EA. Also, we need to explore whether electroacupuncture alleviated morphine tolerance has some relationship with other opioid receptor mechanisms [17].

## Figures and Tables

**Table 1:** Mechanical withdrawal threshold (MWT) of the rats during bone cancer pain and treatment.

	T0	T1	T3	T6	M1	M3	M5	M7	M9
Sham	12.3 ± 2.2	12.4 ± 2.7	12.8 ± 2.2	12.5 ± 2.5	12.3 ± 2.6	12.5 ± 2.5	12.3 ± 2.7	12.2 ± 2.6	12.5 ± 2.2
BM group	12.7 ± 2.8	12.7 ± 2.5	12.4 ± 2.1	8.5 ± 1.0^a^	10.7 ± 0.7^ab^	11.4 ± 2.0^ab^	8.7 ± 0.6^ab^	8.5 ± 2.1^ab^	8.3 ± 2.5^ab^
BME group	12.4 ± 2.4	12.5 ± 2.2	12.5 ± 2.5	8.4 ± 1.8^a^	10.9 ± 0.9^ab^	11.1 ± 2.1^ab^	10.9 ± 0.8^abc^	10.2 ± 1.8^abc^	9.7 ± 2.7^abc^
BE group	12.6 ± 2.5	12.6 ± 2.5	12.3 ± 2.7	8.6 ± 1.8^a^	6.2 ± 0.9^ab^	6.0 ± 1.7^ab^	6.2 ± 1.5^ab^	5.9 ± 1.4^a^	6.1 ± 0.8^a^
B group	12.5 ± 2.2	12.5 ± 2.4	12.4 ± 2.4	8.7 ± 2.0^a^	5.3 ± 1.5^a^	5.1 ± 1.6^a^	5.1 ± 1.5^a^	5.6 ± 1.4^a^	5.7 ± 1.7^a^

^a^Compared with sham, *P* < 0.01; ^b^compared with B group, *P* < 0.01; ^c^compared with BM group, *P* < 0.01.
